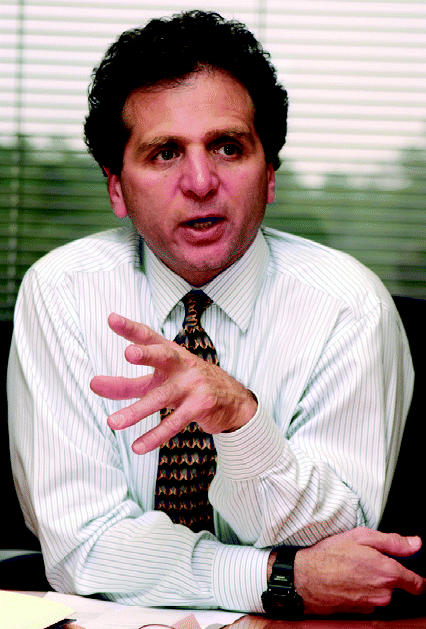# NIEHS Priorities: The Process of Strategic Planning

**DOI:** 10.1289/ehp.113-1257612

**Published:** 2005-06

**Authors:** Sheila Newton, David A. Schwartz

**Affiliations:** Director, Office of Science Policy and Planning, NIEHS; Director, NIEHS E-mail: schwartzd@niehs.nih.gov

As we work to move the NIEHS forward, it is important that we critically consider how our research can have the greatest impact on public health. In last month’s *Director’s Perspective*, I identified my overarching vision for the NIEHS: to improve human health by elucidating the complex relationship between endogenous and exogenous risks within populations and affected individuals, how environmental exposures affect human biology, and how this knowledge can be used to reduce morbidity and extend longevity. Fundamental to this vision is an emphasis on human health and disease. Examples of previous research areas supported by the NIEHS that have had a profound effect on human health include:

- the link between methylmercury and neurodevelopmental deficits in the fetus;
- the role of aflatoxin in the development of hepatocellular carcinoma;
- the importance of manganese in neurodegenerative disorders;
- the adverse developmental, cognitive, cardiovascular, and renal effects of lead;
- the role of polluted air in asthma attacks, and in deaths from chronic obstructive pulmonary disease and cardiovascular disease in the elderly;
- the contribution of paraoxonase to pesticide toxicity;
- the identification of biomarkers of beryllium-induced lung disease; and
- the effect of nitrous oxide on fertility.

If further advances are to be made—and I believe that the environmental health sciences are uniquely poised to profoundly impact the development and progression of disease and substantially improve public health—I am convinced that additional strategic planning is needed to efficiently and effectively focus our resources.

Over the past decades, the NIEHS has periodically conducted outside reviews and assessments of its research directions and priorities, with the objective of setting priorities, readjusting its course, and establishing new goals. The NIEHS has benefited greatly from the input of the nation’s top scientists and experts in environmental health in this process. For instance, the 1970 report *Man’s Health and the Environment* was the result of a task force convened in 1969 by then–NIEHS director Paul Kotin. This document guided some of the early developments of the institute including the development of the environmental health sciences centers. A 1976 report, *Human Health and the Environment*, comprised the input of over 80 scientists and resulted in a comprehensive outline of research needs including focus on specific pollutants of air, food, and water. A 1984 task force report also titled *Human Health and the Environment* focused on how to bring new developments and tools in the biological sciences to bear upon issues in environmental health. In 2000, the NIEHS *Strategic Vision* document outlined the priorities and research objectives of the institute. This document emphasized the importance of disease prevention and placed high priority on efforts targeted towards populations with specific vulnerabilities to environmental insults (minorities, women, and children). It also articulated priorities in new technology development that ultimately matured into what is now termed “toxicogenomics.”

It is timely, then, that we are now embarking on the development of a strategic plan to consider future programmatic development at the NIEHS. Given our anticipated limited resources for the foreseeable future, it is absolutely crucial that we establish our priorities and develop a plan to support the best science that will have the greatest impact on human health.

It is absolutely crucial that we establish our priorities and develop a plan to support the best science that will have the greatest impact on human health.

Throughout the strategic planning process, we will be fully committed to an inclusive approach that draws opinions from a broad array of stakeholders and focuses on critical opportunities in environmental health. To begin the process, we will solicit input from NIEHS-supported investigators and interested stakeholders through the *Federal Register* and the NIEHS web-site (http://www.niehs.nih.gov/). We plan to focus this survey on questions that are critical to the NIEHS:

- What are the disease processes and public health concerns that are relevant to environmental health sciences?
- How can environmental health sciences be used to understand how biological systems work, why some individuals are more susceptible to disease, or why individuals with the same disease have very different clinical outcomes?
- What are the major opportunities and challenges in global environmental health?
- What are the critical exposures that need further investigation?
- What are the critical needs in training the next generation of scientists in environmental health?
- What technology or structural changes are needed to fundamentally advance environmental health sciences?

Responses to these questions will be compiled and analyzed. Then the topics will be discussed in detail by a Strategic Planning Group that will meet sometime this fall. I anticipate that the Strategic Planning Group will comprise approximately 100 individuals, including NIEHS-supported scientists as well as nonscientist stakeholders. The Strategic Planning Group will produce a brief document that outlines the goals and objectives that are thought to warrant the greatest scientific and programmatic attention over the next several years. Once developed, this draft document will be vetted for public comment on the NIEHS website, in the *Federal Register*, and through our advisory councils before a final document is compiled.

In the end, I would like a strategic plan that clearly articulates the goals and objectives that will guide our growth over the next five years. While I am reluctant to commit to a timeline, ideally I would like to have this document completed by early 2006.

I believe that the strategic plan for the next phase of development at the NIEHS will strengthen our institute’s relationship with its constituent communities and create a clear path to meeting the challenges that lie before us. We have unparalleled opportunities to achieve groundbreaking advances in science and to translate these advances into measurable improvements in human health. I am depending on all of you to contribute to the development of the next strategic plan for the NIEHS.

## Figures and Tables

**Figure:**